# Quantitative analysis of *MGMT* promoter methylation in glioblastoma suggests nonlinear prognostic effect

**DOI:** 10.1093/noajnl/vdad115

**Published:** 2023-09-19

**Authors:** David Gibson, Akshay Ravi, Eduardo Rodriguez, Susan Chang, Nancy Oberheim Bush, Jennie Taylor, Jennifer Clarke, David Solomon, Aaron Scheffler, John Witte, Hannah Lambing, Hideho Okada, Mitchel Berger, Farid Chehab, Nicholas A Butowski

**Affiliations:** Department of Neurological Surgery, Division of Neuro-Oncology, University of California, San Francisco, California, USA; Department of Neurological Surgery, Division of Neuro-Oncology, University of California, San Francisco, California, USA; Department of Neurological Surgery, Division of Neuro-Oncology, University of California, San Francisco, California, USA; Department of Neurological Surgery, Division of Neuro-Oncology, University of California, San Francisco, California, USA; Department of Neurological Surgery, Division of Neuro-Oncology, University of California, San Francisco, California, USA; Department of Neurological Surgery, Division of Neuro-Oncology, University of California, San Francisco, California, USA; Department of Neurological Surgery, Division of Neuro-Oncology, University of California, San Francisco, California, USA; Department of Neurological Surgery, Division of Neuro-Oncology, University of California, San Francisco, California, USA; Department of Pathology, University of California, San Francisco, California, USA; Department of Epidemiology and Biostatistics, University of California, San Francisco, California, USA; Department of Epidemiology and Biostatistics, University of California, San Francisco, California, USA; Department of Epidemiology and Biostatistics, University of California, San Francisco, California, USA; Department of Neurological Surgery, Division of Neuro-Oncology, University of California, San Francisco, California, USA; Department of Neurological Surgery, Division of Neuro-Oncology, University of California, San Francisco, California, USA; Institute for Human Genetics, University of California, San Francisco, California, USA; Department of Neurological Surgery, Division of Neuro-Oncology, University of California, San Francisco, California, USA

**Keywords:** bisulfite sequencing, CpG, glioblastoma, methylation, *MGMT*

## Abstract

**Background:**

Epigenetic inhibition of the *O6-methylguanine-DNA-methyltransferase* (*MGMT*) gene has emerged as a clinically relevant prognostic marker in glioblastoma (GBM). Methylation of the *MGMT* promoter has been shown to increase chemotherapy efficacy. While traditionally reported as a binary marker, recent methodological advancements have led to quantitative methods of measuring promoter methylation, providing clearer insight into its functional relationship with survival.

**Methods:**

A CLIA assay and bisulfite sequencing was utilized to develop a quantitative, 17-point, *MGMT* promoter methylation index. GBMs of 240 newly diagnosed patients were sequenced and risk for mortality was assessed. Nonlinearities were captured by fitting splines to Cox proportional hazard models and plotting smoothed residuals. Covariates included age, Karnofsky performance status, *IDH1* mutation, and extent of resection.

**Results:**

Median follow-up time and progression-free survival were 16 and 9 months, respectively. A total of 176 subjects experienced death. A one-unit increase in promoter CpG methylation resulted in a 4% reduction in hazard (95% CI 0.93–0.99, *P* < .005). GBM patients with low levels of promoter methylation (1-6 CpG sites) fared markedly worse (HR = 1.62, 95% CI 1.03–2.54, *P* < .036) than individuals who were unmethylated. Subjects with medium levels of promoter methylation (7–12 sites) had the greatest reduction in hazard (HR = 0.48, 95% CI 0.29–0.80, *P* < .004), followed by individuals in the highest promoter methylation tertile (HR = 0.62, 95% CI 0.40–0.97, *P* < .035).

**Conclusions:**

Our findings suggest that the relationship between the extent of *MGMT* promoter methylation and survival in GBM may be nonlinear. These findings challenge the current understanding of *MGMT* and underlines the clinical importance of determining its prognostic utility. Potential limitations include censoring, sample size, and extraneous mutations.

**Key Points**

- Analysis of quantitative *MGMT* promoter methylation suggests nonlinear prognostic effect.
- Binary representation of *MGMT* promoter methylation may be insufficient for prognostication.

**Importance of the Study**

The following manuscript details a novel laboratory and statistical approach to modeling the nonlinearities of the *MGMT* biomarker in glioblastoma. The results of this study challenge our current understanding of how promoter methylation of the *MGMT* gene effects prognosis and demonstrates the potential downside of binary marker representation. While previous literature suggests that any amount of *MGMT* promoter methylation is beneficial and that the prognostic benefit increases linearly, the results of this analysis bring this current understanding into question. The analysis carried out in this study indicates low-level promoter methylation to prognostically disadvantageous and the highest levels of promoter methylation to be less beneficial than medium levels of promoter methylation. *MGMT* promoter methylation is commonly assessed in glioblastoma patients and treatment regiments are often modulated in response to the results of this marker. For this reason, a clear and quantitative understanding of how the marker shapes prognosis is imperative.

Glioblastoma (GBM) currently represents approximately 50% of high-grade gliomas diagnosed in individuals.^1,2^ Prognostic outlook for GBM is quite poor as it is the most aggressive primary malignant tumor of the central nervous system. Common prognostic indicators of glioblastoma include Karnofsky performance status (KPS), age, extent of tumor resection, radiotherapy, chemotherapy, tumor size, tumor location, and molecular profiling for biomarkers such as *IDH1*, *EGFR*, *PTEN*, and *MGMT*.^1,3,4^ Since the introduction of the 5th edition of the WHO CNS Tumor Classification, IDH-mutant glioblastomas are now referred to as ‘Astrocytoma, IDH-mutant, CNS WHO grade 4.

Highly conserved, the *O*^*6*^*-methylguanine DNA methyltransferase* (*MGMT*) sequence is located at the *10q26.3* position of the 10th chromosome. *MGMT* encodes for the DNA repair protein, *O*^*6*^*-alkylguanine-DNA-alkyltransferase* (*AGT*),^5,6^ which repairs alkylation at the O^6^ position on guanine.^5,7^*AGT* is instrumental in genomic stability and functions by removing alkylating lesions to repair DNA and prevent errors during DNA replication and transcription.^8,6^ This repair process occurs with either open or condensed chromatin, indicating that *AGT* acts independent of target site chromatin remodeling, apart from when its promoter is methylated.^4,5,9,10^ Furthermore, *AGT* does not associate or work in concert with other transcription-coupled repair proteins, indicating *AGT*’s independence as a repair mechanism.^8^

Cells, tissues, and individuals vary greatly in the expression of *AGT*.^5^ The activity of this repair enzyme correlates inversely with sensitivity to agents such as temozolomide, that form *O*^*6*^*-alkylguanine* DNA adducts.^3,5,11^ Expression of the *MGMT* gene is known to play a role in carcinogenesis as a number of malignancies, including gliomas, have increased *MGMT* expression due to lower levels of promoter methylation.^2,5^ When the *MGMT* promoter is unmethylated, the *MGMT* gene is expressed and, therefore, likely to be more effective in repairing DNA that has been damaged by alkylating chemotherapy,^7,8,12^ rendering treatment less effective. This finding is consistent with studies that have shown that tumors with unmethylated *MGMT* promoters have less favorable disease progression and survival outcomes when compared to their methylated counterparts.^9,10,13–18^

*MGMT* promoter methylation is thought to be an independent favorable prognostic marker for improved response to radiation and chemotherapies. Tumors with unmethylated *MGMT* promoters have a less favorable response to alkylating chemotherapeutic agents^4,12,19^ due to *AGT* being actively expressed and repairing the lesions in the DNA caused by treatment.^5,10,20–22^ Epigenetic silencing of the *MGMT* gene has also been shown to be associated with decreased mortality and increased progression-free survival (PFS) and overall survival (OS) when compared to glioblastoma patients with unmethylated *MGMT* promoters.^11,13^

To date, the most prevalent method of testing for *MGMT* promoter methylation, methylation specific PCR (MSP), does not provide quantitative information on the extent of methylation, reporting promoter methylation status as a binary outcome (methylated/unmethylated).^19–21,23^ MSP is commonly utilized due to its efficacy with small Bx samples and relatively low cost when compared to other methods. One of the largest downsides to utilizing MSP is that some weakly or partially methylated tumors are unable to be unequivocally assigned to either a methylated or unmethylated category, rendering the marker uninterperable.^17,24^ To circumnavigate this issue, UCSF currently employs a CLIA-based assay and bisulfite sequencing technique that interrogates 17 distinct CpG sites, generating a quantitative, 17-point index.

While other quantitative methylation techniques are available, methods involving protein expression or activity-based assays run a nonnegligible risk of generating false positives due to nontumor tissue contamination.^17,23,25^ To bypass these pitfalls, alternative methods of analyzing *MGMT* promoter methylation have emerged, including pyrosequencing and high-resolution melt analysis, which have provided promising results and improved discrimination between methylated and unmethylated tumors when compared to MSP.^26–29^The majority of research utilizing such methods, especially MSP, model promoter methylation’s protective effect as a linear relationship and seek to define an optimal cutoff point.^7,11,30,31^ Conversely, a recent study utilizing pyrosequencing detailed that *MGMT* promoter methylation had a nonlinear prognostic relationship where low levels of promoter methylation were inversely associated with survival.^32^ Conflicting descriptions of the association between *MGMT* promoter methylation and survival highlight the importance of further elucidating its protective effect’s functional form. The present study aims to determine how *MGMT* promoter methylation’s advantages in treatment efficacy transform as the extent of promoter CpG silencing increases. By utilizing a novel, methylation assay and various statistical techniques to capture nonlinearities, we further illuminate the functional relationship between *MGMT* promoter methylation and GBM survival.

## Methods

DNA was extracted following macro-dissection of tumor tissue from formalin-fixed paraffin-embedded (FFPE) sections that contained at least 70% tumor cells. DNA was treated with a bisulfite reagent (EZ-DNA Methylation Lightning Kit, Zymo Research, Irvine, CA) followed by two PCR amplification rounds aimed at the synthesis of a 191 bp amplicon (chr10:129,466,812-129,467,002 reference genome GRCh38) with the following forward and reverse PCR primers 5ʹ-ATTATTTTTGTGATAGGAAAAGGTA-3ʹ and 5ʹ-AAACAATCTACGCATCCT-3ʹ. The 191 bp amplicon spans 17 CpG sites and 31 nonCpG cytosines that serve as internal controls for the efficiency of bisulfite treatment. The 191 bp amplicon includes 17 CpG sites with the first CpG site located 228 bp upstream of rs2782888. CpGs 1-5 are located 85 bp upstream of DMR1 and CpGs 6-17 extend 106 bp within DMR1. Each PCR round consisted of 35 cycles at 95^o^C for 15 s, 54^o^C for 30 s, and 72^o^C for 30 s. In the second PCR round, a 1 μl aliquot from the first PCR is used in each of 2 amplification reactions where either the forward or reverse PCR primer is biotinylated. After the second PCR, the reactions are purified with streptavidin magnetic beads and subjected to Sanger Sequencing on an Applied Biosystems 3130 instrument. To control for PCR contamination, each of the 2 rounds of PCR utilizes an FFPE DNA extraction blank control. Prior to sequencing, these blank controls are run on an agarose gel along with the amplification reactions to ensure appropriate amplification of the targeted 191 bp amplicon without any amplification in the blank controls. The DNA sequencing reactions from each biotinylated primer were performed in duplicate, yielding a total of 4 overlapping DNA sequencing reactions. The sequences were assembled and visualized using CodonCode Aligner (CodonCode Corporation, Centerville MA). All 31 cytosine control sites are required to be converted to thymines before a methylation index, ranging from 0 to 17, is determined. Our Sanger bisulfite sequencing assay does not quantify the extent of promoter methylation at individual CpG sites. Rather, it detects the methylation of a CpG site as denoted by the presence of a cytosine nucleotide, within the sensitivity of Sanger DNA sequencing (approximately 10%). The total number of methylated CpG sites is reported as the methylation index. An index score of 0 corresponds to an unmethylated *MGMT* promoter, whereas an index score ranging from 1 to 17 corresponds to a methylated *MGMT* promoter. A one-unit increase in promoter methylation index score corresponds to an additional CpG site being methylated.

Prior to surgery, all subjects signed consent for the use of their tissue, demographic information, and clinical information obtained through query of electronic medical records. This tissue consent was approved by the UCSF institutional Review Board. The inclusion criteria defined for this study were adult patients (≥18 years of age) with glioblastoma (WHO grade IV) centered in the cerebral hemispheres, who underwent surgical intervention at UCSF, including biopsy (Bx), subtotal resection (STR), or gross total resection (GTR) and received standard adjuvant radiation/chemotherapy. Tumor samples acquired from UCSF patients with newly diagnosed GBM were analyzed using the previously described assay. Promoter methylation results from 300 patients’ FFPE tumor samples collected during initial surgical resection between 2009 and 2017 were evaluated and followed for survival until 2020. Data cleaning procedures where subjects with lower grade gliomas, nonprimary GBM cases, and cases with no follow-up data (less than 2 weeks) were excluded, yielded a final cohort of 240 subjects. Median follow-up time for OS was 16 months (IQR 21.9).

Data on age, gender, KPS, extent of resection, location of tumor, time to recurrence, time to death (or last follow-up), *isocitrate dehydrogenase 1* mutation status (*IDH1*-*R132H*), and methylation index score were retrospectively collected. Extent of resection was defined by neuroradiology in conjunction with the UCSF tumor board and based on a comparison of pre and postoperative contrast imaging.

Data cleaning, wrangling, and analysis were performed using R software (version 4.0.2). Cox Proportional Hazard models were constructed with methylation index results of patients in continuous and ordinal categorical (methylation tertiles and quintiles) formats. Models were adjusted for age, KPS, extent of resection, and *IDH1* mutation. The statistical significance level was 0.05. Multivariate Cox models were then utilized to create survival plots. To capture nonlinearities, basis splines, P-splines, and restricted cubic splines were fit to the aforementioned Cox models. To test model fit and to further assess the functional form of *MGMT* promoter methylation, we plotted the smoothed Martingale residuals of an unadjusted, Cox proportional hazard model. The assumption of proportionality was tested by plotting Schoenfeld residuals against the transformed time.

## Results

### Patient Demographics

Patient baseline characteristics parsed out by methylation tertile are listed in Table 1. Overall median age was 60 (IQR 44–76) with 59% of all subjects being male and 39% being female. Karnofsky performance status at time of resection was reported by 80% of patients with the median score being 80 (IQR 70–90). GTR was achieved in 44% of patients, STR was achieved in 31% of patients, while 14% of patients only had a Bx. Data on extent of resection was unavailable in 11% of subjects. A total of 64% of patients’ tumors exhibited *MGMT* promoter methylation. The median number of methylated CpG sites (index score) was 2 (IQR 0–12). Of the patients with methylated tumors, the median index score was 8. *IDH1-R123H* mutation data was available for 92% of patients with 15 patients (6%) testing positive.

**Table 1.** Clinical Characteristics at Baseline (*n* = 240).

	Unmethylated MGMT (0 CpG Sites) (*N* = 115)	Low Level Methylation (1–6) (*N* = 44)	Medium Level Methylation (7–12) (*N* = 38)	High Level Methylation (13–17) (*N* = 43)
Age				
Mean (SD)	58.5 (11.5)	57.4 (14.8)	61.0 (11.8)	60.0 (12.2)
Median [Min, Max]	59.0 [23.0, 82.0]	56.0 [21.0, 83.0]	62.5 [35.0, 81.0]	62.0 [33.0, 81.0]
Gender				
Male	73 (63.5%)	22 (50.0%)	23 (60.5%)	24 (55.8%)
Female	42 (36.5%)	22 (50.0%)	15 (39.5%)	19 (44.2%)
Death				
Censored	47 (40.9%)	7 (15.9%)	8 (21.1%)	3 (7.0%)
Experienced event	68 (59.1%)	37 (84.1%)	30 (78.9%)	40 (93.0%)
Overall survival (months)				
Mean (SD)	18.8 (18.0)	20.8 (18.0)	32.4 (29.2)	34.0 (27.6)
Median (min, max)	14.0 [0.53, 88]	15.46 [3.27, 91.3]	25.7 [0.51, 125.7]	27.47 [0.97, 131.7]
Time to disease progression (months)				
Mean (SD)	11.6 (11.1)	10.4 (5.8)	25.3 (22.7)	22.0 (20.6)
Median (min, max)	8 [0.83, 52.3]	10 [1.0, 27]	20 [1.4, 100]	17.5 [1.2, 107.3]
Extent of resection				
Biopsy	19 (16.5%)	6 (13.6%)	6 (15.8%)	3 (7.0%)
Subtotal resection	37 (32.2%)	11 (25.0%)	14 (36.8%)	13 (30.2%)
Gross total resection	48 (41.7%)	21 (47.7%)	14 (36.8%)	23 (53.5%)
Karnofsky performance status				
Mean (SD)	82.0 (10.5)	82.1 (10.8)	78.5 (8.57)	81.3 (11.0)
Median (min, max)	80.0 [50.0, 100]	80.0 [50.0, 100]	80.0 [60.0, 90.0]	80.0 [40.0, 100]
IDH-1 mutation				
Wild type	103 (89.6%)	34 (77.3%)	33 (86.8%)	36 (83.7%)
Mutated	5 (4.3%)	2 (4.5%)	4 (10.5%)	4 (9.3%)
EGFR gene				
Wild type	60 (52.2%)	17 (38.6%)	17 (44.7%)	13 (30.2%)
Amplified	44 (38.3%)	18 (40.9%)	17 (44.7%)	20 (46.5%)
PTEN gene				
Wild type	28 (24.3%)	10 (22.7%)	12 (31.6%)	13 (30.2%)
Deleted	70 (60.9%)	25 (56.8%)	20 (52.6%)	21 (48.8%)
Tumor protein P53				
Negative	15 (13.0%)	6 (13.6%)	7 (18.4%)	5 (11.6%)
Positive in cells	63 (54.8%)	18 (40.9%)	15 (39.5%)	19 (44.2%)

Bolded values denote statistical significance at an alpha level of .05. In other words in at least 19/20 cases we hold the estimates of our regression model to be true.

### Overall Survival

Total median follow-up time was 16 months, during which 176 subjects experienced death. Median OS for unmethylated subjects was 14 months. OS increased linearly with extent of promoter methylation where low-level promoter methylation (1–6 CPG sites) had a median OS of 15.4 months, medium-level methylated subjects (7–12 CpG sites) had a median OS of 25.7 months, and high-level promoter methylation (13–17 CpG sites) had a median OS of 27.5 months. Summarized in Table 2, Cox proportional hazards models were utilized when assessing how OS differed as extent of *MGMT* promoter methylation increased. Despite median OS increasing in a uniform fashion as promoter methylation increases (Table 1), hazard ratios for promoter methylation transformed in a nonlinear pattern. When considering nonmethylated subjects as the reference group and adjusting for age, KPS, and *IDH1* mutation, our model demonstrated a 4% reduction in hazard (95% CI 0.93–0.99, *P* < .005) for every one-unit increase in promoter methylation index. Modeling promoter methylation status as a binary marker resulted in methylated subjects experiencing a 40% reduction of risk for mortality (HR = 0.60 95% CI 0.41–0.87, *P* = .008). Moreover, GBM patients with low levels of promoter methylation fared markedly worse (HR = 1.62, 95% CI 1.03–2.54, *P* < .036) than individuals who were unmethylated. Subjects with medium levels of promoter methylation had the greatest reduction in hazard (HR = 0.48, 95% CI 0.29–0.80, *P* < 0.004), followed by individuals in the highest methylation tertile (HR = 0.62, 95% CI 0.40–0.97, *P* < .035). Our Cox proportional hazard model had a Concordance index of 0.66 (SE = 0.025).

**Table 2.** Summary Statistics for Overall Survival, Cox Proportional Hazard Models (*N* = 240).

Model	Variable	HR	95% CI	*P-*value	Test	Test Statistic	DF	*P-*value
Binary								
	Unmethylated (*n = 85)*	Reference	Reference	Reference	LR test	18.66	4	**9e−04**
	Methylated (*n* = 155)	0.79	0.54–1.2	0.232	Wald test	16.11	4	**0.003**
					Logrank test	16.79	4	**0.002**
	Age	1.01	1.0–1.0	0.113	Concordance (SE)	0.61 (0.027)		
	KPS	0.98	0.97–1.0	0.061				
	IDH1	0.46	0.24–0.9	**0.023**				
Tertile								
	Unmethylated (*n* = 115)	Reference	Reference	Reference	LR test	36.8	6	**2e−06**
	1–6 CpG sites (*n* = 44)	1.62	1.03–2.54	**0.036**	Wald test	33.89	6	**7e−06**
	7–12 CpG sites (*n* = 38)	0.48	0.29–0.80	**0.004**	Logrank test	35.29	6	**4e−06**
	13–17 CpG sites (*n* = 43)	0.62	0.40–0.97	**0.04**	Concordance (SE)	0.66 (0.025)		
								
	Age	1.02	1.01–1.04	**0.0006**				
	KPS	0.98	0.96–0.99	**0.005**				
	IDH1	0.56	0.28–1.12	0.1				
Quintile								
	Unmethylated (*n* = 115)	Reference	Reference	Reference	LR test	39.44	8	**4e−06**
	1–3 CpG sites (*n* = 24)	1.99	1.16–3.43	**0.013**	Wald test	36.51	8	**1e−05**
	4–6 CpG sites (*n* = 20)	1.28	0.68–2.40	0.445	Logrank test	37.94	8	**8e−06**
	7–9 CpG sites (*n* = 14)	0.46	0.22–0.95	**0.035**	Concordance (SE)	0.66 (0.026)		
	10–13 CpG sites (*n* = 31)	0.46	0.27–0.78	**0.004**				
	14–17 CpG sites (*n* = 36)	0.68	0.43- 1.09	0.108				
								
	Age	0.98	0.96–0.99	**0.005**				
	KPS	1.02	1.01–1.04	**0.004**				
	IDH1	0.55	0.27–1.10	0.091				
Continuous								
	0–17 CpG sites (*n* = 240)	0.96	0.93–0.99	**0.005**	LR test	25.37	4	**4e−05**
					Wald test	22.6	4	**2e−04**
	Age	1.02	1.0–1.03	0.042	Logrank test	23.47	4	**1e−04**
	KPS	0.98	0.96–1.0	**0.029**	Concordance (SE)	0.64 (0.027)		
	IDH1	0.50	0.25–0.98	**0.044**				

Bolded significance values with regard to IDH-1 denote a significant difference between IDH-1 WT and mutant groups. Bolded significance values with regard to Binary, Tertile, and Quintile MGMT promoter methylation groups denote significant difference when compared to the references, non-methylated MGMT promoter group. A significant *P*-value from the likelihood ratio test indicates that the inclusion of MGMT promoter methylated groups differ significantly with regard to survival than a model that includes only the reference group. Significant *P*-values from the Wald test suggest that the extent of MGMT promoter methylation is a significant predictor in the regression model, while a significant *P*-value from the log-rank test implies differences in survival experiences between the MGMT promoter methylation groups.

To test the assumption of proportionality, we plotted scaled Schoenfeld residuals corresponding to different variables included in the Cox model to determine if the residuals increased or decreased over time (indicating departure from the proportionality assumption). We determined that there was no significant interaction effect between time and promoter methylation (Supplementary Figure S2) as the *P*-value was 0.15 (Chi-square = 2.06 on 1 df). In this case, a significant *P*-value indicates that the proportional hazards assumption is violated.

**Figure 1.**
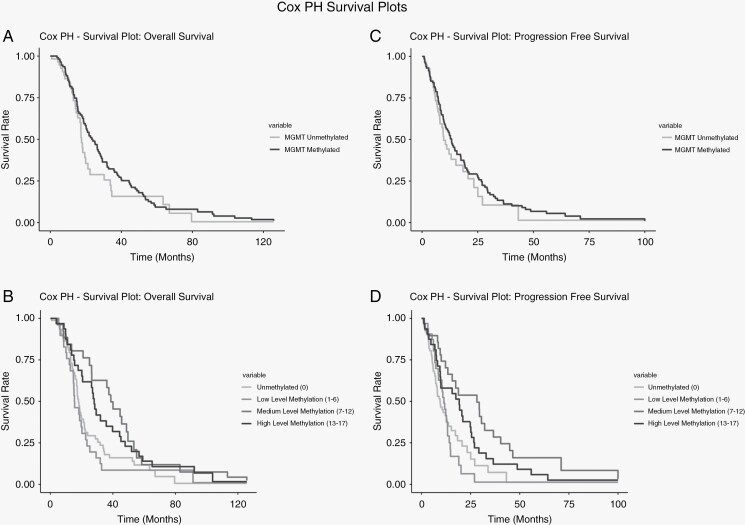
(A) Cox PH survival plot for binary methylation status. (B) Cox PH survival plot for methylation tertiles. (C) Cox PH progression-free survival (PFS) plot for binary methylation status. (D) Cox PH PFS plot for methylation tertiles. All models were adjusted for KPS, age, extent of resection, and IDH1 status.

We reject the null hypothesis in favor of the full as our model reported an estimated likelihood ratio of 36.8 on 6 df (*P* = 2e−06), a Wald Chi-square statistic of 33.89 on 6 df (*P* = 7e−06), and a (Logrank) test = 35.29 on 6 df (*P* = 4e−06). There is a significant difference between different promoter methylation groups, index scores, and status (methylated/unmethylated). The difference between the ordinal categorical levels of promoter methylation is visually apparent in the survival curves illustrated in Figure 1. These survival curves are plotted as a Cox function of OS and promoter methylation category and are adjusted by the covariates; extent of resection, KPS, age, and *IDH-1* mutation status.

**Figure 2.**
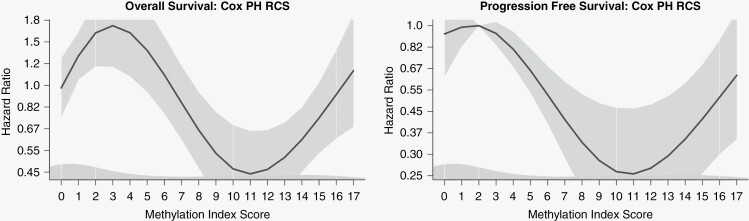
Restricted cubic splines (RCS) with 5 knots and 3 df fitted to Cox proportional hazard models that are adjusted for KPS, age, extent of resection, and IDH1 status. This plot is a graphical representation of how our Cox model’s estimate hazard for mortality or disease progression as methylation increases. An index score of 0 equates to having no MGMT promoter CpG sites methylated. The RCS for the overall survival model has a Chi-square of 116.6 (*P* < 2e−16). While the RCS for PFS had a Chi-square of 6.47 (*P* = .091).

When modeling promoter methylation index score as a continuous variable we utilized splines to capture nonlinearities in its relationship with OS. As indicated in Figure 2, the restricted cubic spline (RCS) fitted to our Cox model indicated conformation of promoter methylation’s prognostic advantage. Subjects who had between 9 and 13 CpG sites methylated had the greatest reduction in hazard, while the lower levels of promoter methylation had a significant increase in hazard over the unmethylated group. The smoothed curve falls well within the appropriate 95% confidence intervals (Chi-square 116.6, 3 df, *P* < 2e−16) and approximates the form of both the survival curves and ordinal categorical Cox model. Alternate spline models including P-splines and B-splines demonstrated the same shape or functional form of the relationship between *MGMT* promoter methylation and OS. All spline models contained the same covariates as the previously mentioned survival plot in Figure 1. We found that the unadjusted Martingale residuals for the continuous promoter methylation index’s Cox model displayed nonlinearity in a manner similar to the spline models (Figure 2).

### Progression-Free Survival

The median time to radiographic disease progression was 9 months. Unmethylated subjects had a median PFS time of 8 months, low-level methylated subjects (1–6 CpG sites) had a median PFS of 9.7 months, medium levels of promoter methylation (7–12 CpG sites) had a median PFS of 19.9 months, and highly methylated subjects (13–17 CpG sites) had a median PFS of 17.5 months. The Cox PH forest plot in Table 3 is a graphical display of the estimates from a PFS model for promoter methylation, adjusting for age, *IDH-1* mutation, and extent of resection. Subjects with low levels of promoter methylation (1–6) showed no significant change in risk (HR = 0.85, 95% CI 0.53–1.36, *P* = .496) when compared to subjects with unmethylated tumors. Subjects with medium levels of promoter methylation (7–12) showed a 73% reduction in risk (HR = 0.27, 95% CI 0.16–0.46, *P* < 0.001) whereas subjects with highly methylated tumors (13–17) had a 54% reduction in risk for mortality (HR = 0.46, 95% CI 0.29–0.72, *P* < .001). Differences in time to radiographic disease progression between levels of promoter methylation are visualized in the survival plot in Figure 1. Survival plot utilizes estimates from a Cox proportional hazard model adjusted for extent of resection, age, KPS score, and *IDH1* mutation status. Additional illustrations for the PFS endpoint can be found in the supplemental materials section.

**Table 3.** Summary Statistics for Progression-Free Survival, Cox Proportional Hazard Models (*N* = 240).

Model	Variable	HR	95% CI	*P*-Value	H Test	Test Statistic	DF	*P-V*alue
Binary								
	Unmethylated (*n* = 85)	Reference	Reference	Reference	LR test	14.64	6	**0.02**
	Methylated (*n* = 155)	0.6	0.41–0.87	**0.008**	Wald test	14.62	6	**0.02**
					Logrank test	14.89	6	**0.02**
	Age	0.99	0.98–1.01	0.5	Concordance (SE)	0.60 (0.026)		
	KPS	0.99	0.97–1.01	0.25				
	IDH1	0.46	0.22–0.97	**0.04**				
	Resection: Bx	Reference	Reference	Reference				
	STR	1.96	1.01–3.80	**0.05**				
	GTR	1.66	0.87–3.19	0.13				
Tertile								
	Unmethylated (*n* = 115)	Reference	Reference	Reference	LR test	36.96	8	**1e−05**
	1–6 CpG sites (*n* = 44)	0.85	0.53–1.36	0.5	Wald test	33.27	8	**6e−05**
	7–12 CpG sites (*n* = 38)	0.27	0.16–0.46	**1.5e-06**	Logrank test	35.16	8	**3e−05**
	13–17 CpG sites (*n* = 43)	0.46	0.29–0.72	**0.0008**	Concordance (SE)	0.63 (0.027)		
								
	Age	1.00	0.97–1.00	0.988				
	KPS	0.99	0.98–1.02	0.105				
	IDH1	0.52	0.24–1.08	0.081				
	Resection: Bx	Reference	Reference	Reference				
	STR	2.08	1.06–4.08	**0.034**				
	GTR	1.59	0.82–3.08	0.169				
Quintile								
	Unmethylated (*n* = 115)	Reference	Reference	Reference	LR test	37.57	10	**5e−05**
	1–3 CpG sites (*n* = 24)	0.8	0.45–1.43	0.45	Wald test	33.38	10	**2e−04**
	4–6 CpG sites (*n* = 20)	0.92	0.47–1.81	0.81	Logrank test	35.38	10	**1e−04**
	7–9 CpG sites (*n* = 14)	0.21	0.09–0.52	**0.001**	Concordance (SE)	0.63 (0.027)		
	10–13 CpG sites (*n* = 31)	0.31	0.18–0.53	**74e−05**				
	14–17 CpG sites (*n* = 36)	0.48	0.29–0.78	**0.003**				
								
	Age	1.00	0.98–1.02	0.96				
	KPS	0.99	0.97–1.00	0.113				
	IDH1	0.54	0.25–1.16	0.115				
	Resection: Bx	Reference	Reference	Reference				
	STR	1.94	0.99–3.81	**0.05**				
	GTR	1.46	0.75–2.83	0.26				
Continuous								
	0–17 CpG sites (*n* = 240)	0.94	0.91–0.97	**7.21e-05**	LR test	24.77	6	**4e−04**
					Wald test	23.01	6	**8e−04**
	Age	1.00	0.98–1.01	0.73	Logrank test	23.61	6	**6e−04**
	KPS	0.99	0.97–1.01	0.19	Concordance (SE)	0.62 (0.027)		
	IDH1	0.47	0.22–0.99	**0.05**				
	Resection: Bx	Reference	Reference	Reference				
	STR	1.93	0.99–3.77	**0.05**				
	GTR	1.59	0.83–3.04	0.16				

Bolded significance values with regard to IDH-1 denote a significant difference between IDH-1 WT and mutant groups. Bolded significance values with regard to Binary, Tertile, and Quintile MGMT promoter methylation groups denote significant difference when compared to the references, non-methylated MGMT promoter group. A significant *P*-value from the likelihood ratio test indicates that the inclusion of MGMT promoter methylated groups differ significantly with regard to survival than a model that includes only the reference group. Significant *P*-values from the Wald test suggest that the extent of MGMT promoter methylation is a significant predictor in the regression model, while a significant *P*-value from the log-rank test implies differences in survival experiences between the MGMT promoter methylation groups.

## Discussion

In this study, we utilize a novel *MGMT* promoter methylation index to determine how the extent of promoter methylation effects OS and time to disease progression. While *MGMT* promoter methylation’s relationship with survival has traditionally been described as linear,^30,33,34^ our data contrasts this, indicating that the prognostic effect of *MGMT* promoter methylation follows a pattern of nonlinear conformation. We found that low-level promoter methylation carried a significantly higher risk for mortality than being unmethylated. Furthermore, our data indicates that *MGMT* promoter methylation offers the greatest prognostic benefits when 9–13 CpG sites exhibit methylation, as subjects with medium levels of promoter methylation had a lower risk for mortality when compared to subjects who were highly methylated. These findings suggest that a cutoff point or threshold may not be optimal in delineating how a patient will perform based on their level of promoter methylation.

A recent study utilizing a quantitative pyrosequencing approach conducted by Giuseppe et al. concluded that methylation at less than 15% of the CpG sites they interrogated was associated with impaired survival (HR 2.7 95% CI 2.1–3.4 *P* < .00001).^13^ In our study we also found that low levels of promoter methylation were associated with increased risk for mortality. When stratifying our 17-point methylation index into quintiles, subjects who had 1–3 CpG sites methylated had a 100% increase in hazard. (HR 1.99, 95% CI 1.16–3.43, *P* < 0.013). We expected that the benefits of *MGMT* promoter methylation would follow a linear trend where the highest levels of promoter methylation offered the greatest prognostic benefits. Surprisingly, our data exhibited a pattern of conformation where subjects with medium levels of promoter methylation had a greater reduction in risk than those with the highest levels of promoter methylation. This pattern of nonlinear conformation was also present for the PFS outcome; however, we did not find a statistically significant difference between lower levels of promoter methylation and the unmethylated reference group.

The functional form of our promoter methylation index, which was captured through the use of splines and smoothed martingale residuals, is likely the result of multiple factors acting in unison. Smoothed martingale residuals are a way of assessing the goodness-of-fit of Cox proportional hazard models that are smoothed using kernel density estimation. The residuals are calculated from piecewise polynomial models (splines) that model nonlinearity. While it is possible this trend in hazard ratio may be due in part to sample size, these findings are consistent with those of Giuseppe et al., which had a total enrollment of 681 patients. Our OS Cox model did not include the extent of resection as a covariate, as including extent of resection did not change the shape of how the estimated hazard ratio transformed over an increase in promoter methylation index score. Extent of resection was left out of this model as it increased the estimated *P*-values but did not significantly change the estimated hazard ratios. However, extent of resection was included as a covariate in the survival plots and spline models.

The analysis presented in this study includes two important measurements: median OS and hazard ratio. Median OS is a measure of central tendency, reflecting the time point at which half of the population has died. In contrast, HRs represent the adjusted relative risk of an event occurring in one promoter methylation group compared to another group. It is important to note that the trends between these two measurements differ as they measure different aspects of survival.

It remains unclear as to what may be responsible for causing the low-level promoter methylation group’s large increase in hazard. It is possible that subjects who had lower levels of promoter methylation were barred from or wrongly permitted to participate in certain investigation interventions upon recurrence, affecting survival. This may be due in part to a potential “gray zone.” As initially discussed by Wick et al., the “gray zone” represents weakly or partly methylated tumors that often cannot be unequivocally assigned to the methylated or unmethylated categories.^12^ Several ideas have been proposed to better explain this “gray zone,” including heterogeneous methylation patterns across the *MGMT* CpG islands, as well as a biochemical challenge in adequately distinguishing between methylated and unmethylated in tumor tissue with variable contamination of benign tissue.^12^ In several instances, subjects who progressed and went on to have subsequent surgeries would send out tissue for central review as part of the inclusion/exclusion criterium of a clinical trial. We found that in some cases, subjects with low promoter methylation index scores (1–3) would be considered unmethylated by a central lab’s MS-PCR analysis, affecting the patient’s eligibility for enrollment in an investigational therapy.

The apparent increase in risk from medium to high-level promoter methylation may be due in part to small sample sizes. Outlying long-term survivors (LTS) may skew the medium level of the promoter methylation group’s estimated hazard ratio. Eighteen patients had a time to last follow-up greater than 3 years. Of these 18 long term survivors, 2 were unmethylated, 2 had low levels of promoter methylation (1 CpG site), 8 exhibited medium levels of promoter methylation (9–10 sites), and 6 were highly methylated (13–16 sites). A follow-up study with a larger sample size would be necessary to verify whether the presence of LTS outliers is affecting the shape of our data.

Recent research has found that temozolomide induces somatic hypermutation, leading to transformation of grades.^35^ Upon recurrence, these TMZ-treated hypermutated tumors harbored driver mutations in key tumor suppressors and oncogenes.^36^ It is quite likely that temozolomide increases tumor mutational load in primary GBM as well^37^, affecting OS and time to disease progression. *MGMT* promoter methylation increases temozolomide efficacy, thus having high levels of promoter methylation may correlate with increased intertumoral mutation.^35^ Hypermutated gliomas are more resistant to alkylating chemotherapies and harbor unique molecular vulnerabilities such as greater expression of mutant neoantigens on their cell surfaces.^36^ The conformation of promoter methylation’s prognostic effect found in our data may represent an optimal level of *AGT* inhibition where the repair enzyme is able to mitigate some of temozolomide’s cytotoxic effects by repairing mutagenic DNA lesions while still rendering the chemo alkylating agent more effective than it would be under lower levels of promoter methylation.

There are a number of additional factors to take into consideration when interpreting the estimates from our models. For one, glioblastoma tumors are cellularly and molecularly heterogeneous, both within and between tumors.^36^ To this extent, *MGMT* promoter methylation may vary within samples of a single lesion, different multi-focal lesions, or between reoccurrences.^38^ This variability may be a source of sampling bias that is unaccounted for. In addition to tumor heterogeneity, left and right censoring may affect model estimates and survival curves. We have no clear way of inferring how long a patient was living with GBM before time of diagnosis, and only 176 out of 240 subjects experienced death, several were lost to follow-up. It also remains unclear how important a predictor *MGMT* promoter methylation is as a stand-alone prognostic marker. Despite including *IDH-1* mutated patients (*n* = 15) in our models, we adjusted for *IDH-1* status as a covariate. We found that excluding *IDH-1* patients did not significantly affect model estimates or the functional form of our regressor’s relationship with OS.

A potential confounding factor in all *MGMT* promoter methylation assays is the mixed extent of methylation at each individual CpG sites. Each CpG island varies in how heavily methylated the site is. For this reason, we did not calculate the methylation percent of a methylated CpG site, but simply scored it as methylated when a cytosine nucleotide was detected on a background of fully converted 31 CpG control sites. We controlled for the integrity of the bisulfite treatment by ensuring the conversion of the 31 control sites before calculating the methylation index score. Finally, the possibility of point mutations introduced during the amplification process by DNA polymerase errors was controlled by the use of a Taq DNA polymerase with a proofreading function, as well as subsequent monitoring of the aberrant nucleotides noted by the analysis software.

*MGMT* promoter methylation in GBM has been found to be both a prognostic and predictive marker. While it is well-established that *MGMT* promoter methylation leads to improved response to radiation and chemotherapy, it is also possible that *MGMT* silencing may be a pretumorigenic mechanism. Studies have shown that *MGMT* silencing is linked to mutations in other tumor-related genes, such as *p53*, *k-ras*, and *CDKN1A/2A*, which are markers of poor prognosis.^8^ Although the relationship between methylation of genes in carcinogenesis is not entirely clear, these findings suggest that *MGMT* promoter methylation likely has an impact on GBM tumor biology beyond treatment resistance.

Our findings suggest that making treatment decisions guided by an “optimal methylation cut-off point,” as suggested by other studies, may carry inherent risks and negative clinical implications. When interpreting quantitative promoter methylation testing results, it is imperative that clinicians consider that the functional form of the relationship between promoter methylation and OS may not be linear. To validate and build upon the findings of this study, we intend to conduct research utilizing next-generation sequencing data from a larger sample in order to derive how the extent of methylation of each individual CpG site and their locations within the promoter region of the *MGMT* gene affect the marker’s prognostic performance. Research of this nature will assist to further characterizing the inherent qualities of the relationship between *MGMT* promoter methylation and survival in glioblastoma.

## Supplementary Material

vdad115_suppl_Supplementary_Figure_S1

vdad115_suppl_Supplementary_Figure_S2

vdad115_suppl_Supplementary_Table
