# Supplementary figures and images for: Quantitative analysis of MGMT promoter methylation in glioblastoma suggests nonlinear prognostic effect
Source: Neurooncol Adv. 2023 Sep 19;5(1):vdad115. doi: 10.1093/noajnl/vdad115 (PMC10611422; doi:10.1093/noajnl/vdad115)

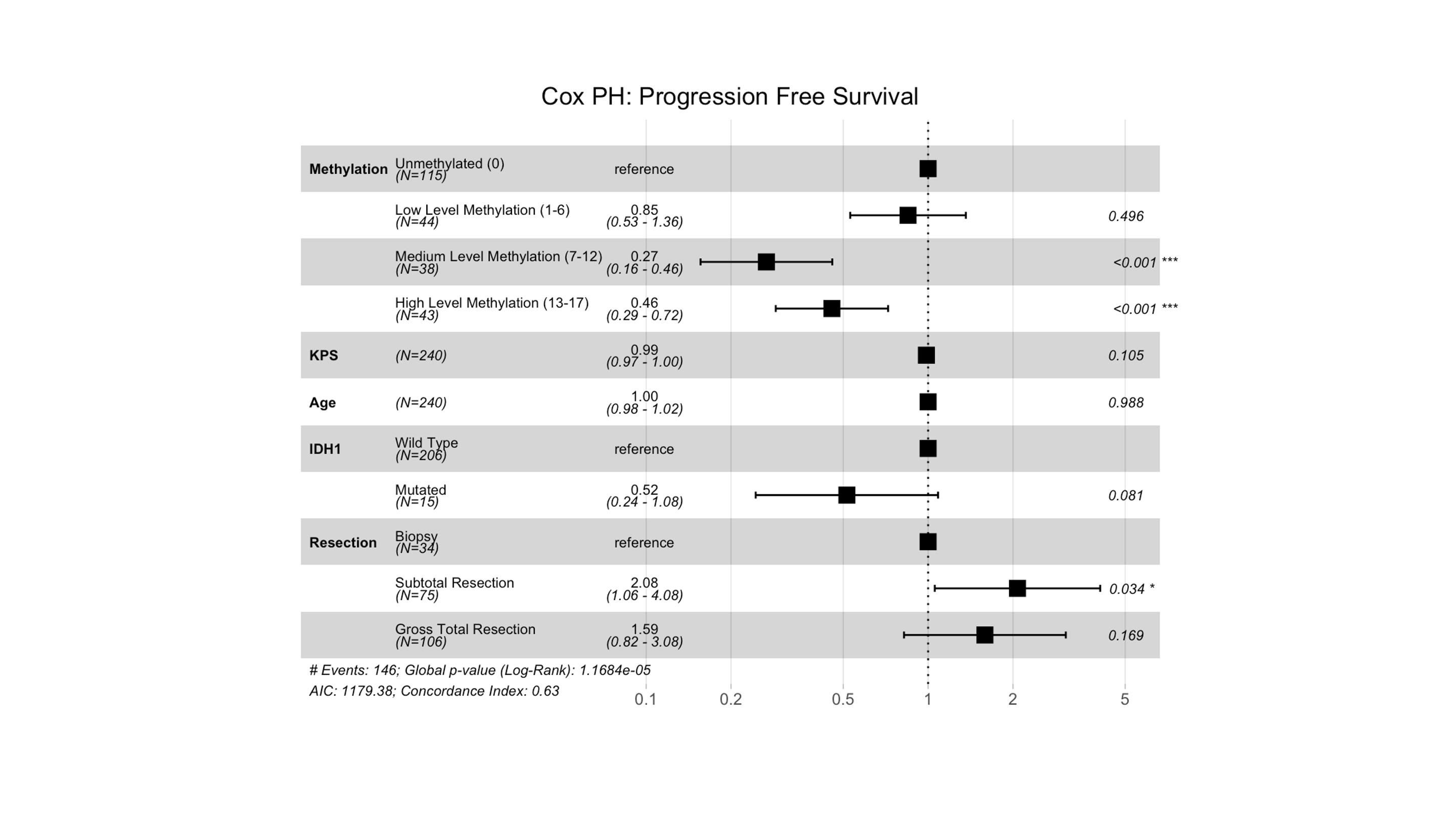

Supplement: vdad115_suppl_Supplementary_Figure_S1 [file vdad115_suppl_supplementary_figure_s1.jpeg]

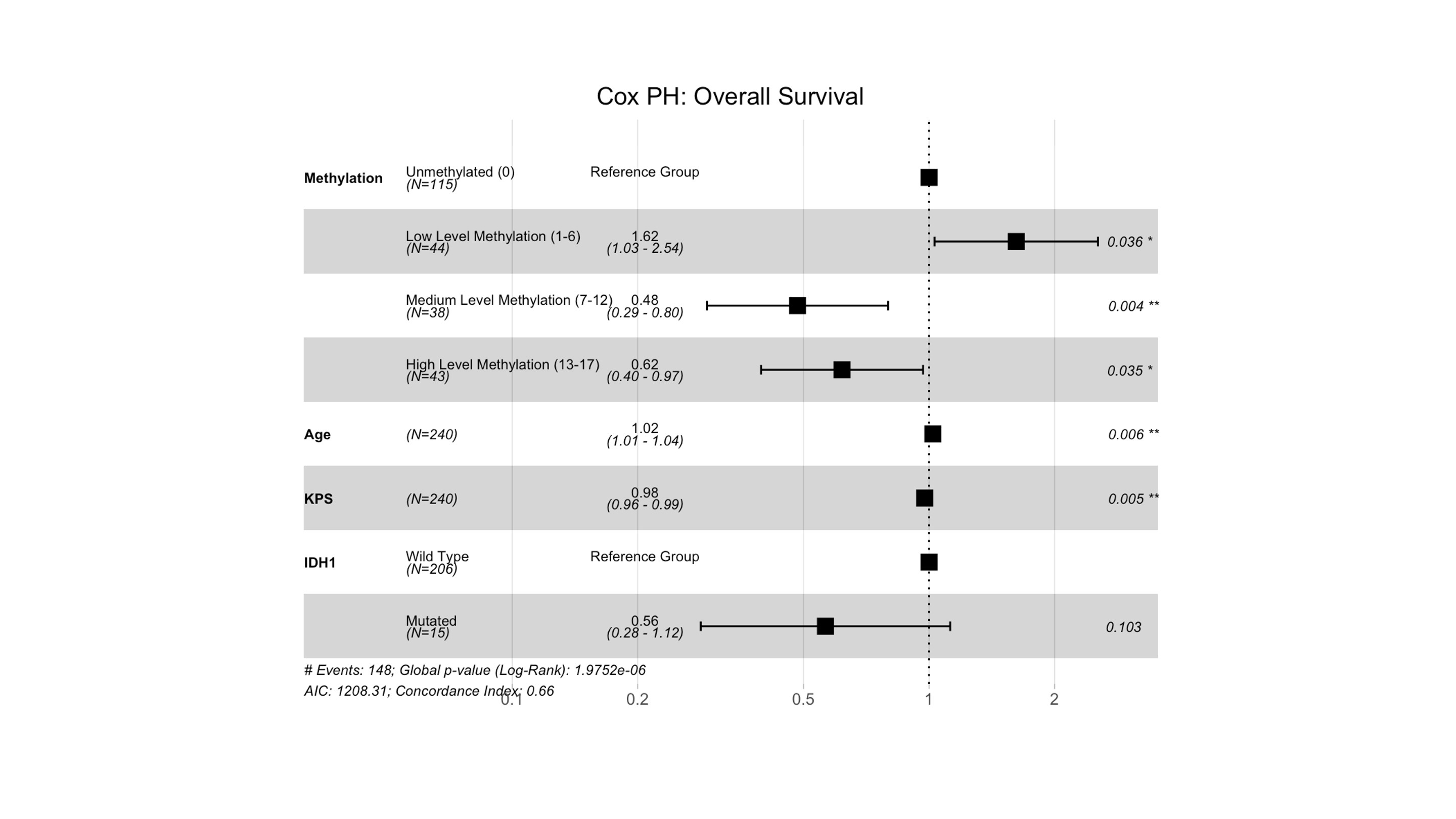

Supplement: vdad115_suppl_Supplementary_Figure_S2 [file vdad115_suppl_supplementary_figure_s2.jpeg]
