# Supplementary material for: Quantitative analysis of MGMT promoter methylation in glioblastoma suggests nonlinear prognostic effect
Source: Neurooncol Adv. 2023 Sep 19;5(1):vdad115. doi: 10.1093/noajnl/vdad115 (PMC10611422; doi:10.1093/noajnl/vdad115)
Supplement: vdad115_suppl_Supplementary_Table [file vdad115_suppl_supplementary_table.docx]

Supplemental Table 1. Locations of CpG sites used for *MGMT* methylation score

| # | CpG site coordinates* | Position relative to Transcription Start Site | CpG # in chronological order for *MGMT* gene |
| --- | --- | --- | --- |
| 1 | chr10:131,265,101 | -347 | 21 |
| 2 | chr10:131,265,137 | -311 | 22 |
| 3 | chr10:131,265,152 | -296 | 23 |
| 4 | chr10:131,265,155 | -293 | 24 |
| 5 | chr10:131,265,159 | -289 | 25 |
| 6 | chr10:131,265,169 | -279 | 26 |
| 7 | chr10:131,265,173 | -275 | 27 |
| 8 | chr10:131,265,185 | -263 | 28 |
| 9 | chr10:131,265,194 | -254 | 29 |
| 10 | chr10:131,265,206 | -242 | 30 |
| 11 | chr10:131,265,209 | -239 | 31 |
| 12 | chr10:131,265,215 | -233 | 32 |
| 13 | chr10:131,265,229 | -219 | 33 |
| 14 | chr10:131,265,232 | -216 | 34 |
| 15 | chr10:131,265,237 | -211 | 35 |
| 16 | chr10:131,265,240 | -208 | 36 |
| 17 | chr10:131,265,247 | -201 | 37 |

The position of CpG sites interrogated in *MGMT* methylation index assay correlated to site coordinates, position relative to transcription start site, and the number of the CpG site often used to discuss *MGMT* promoter methylation. *Based on hg19 human genome assembly.
